# Association Between Acute COVID‐19 Infection and Long COVID in a Non‐Hospitalized Population: A Retrospective Case‐Control Study

**DOI:** 10.1002/hsr2.71043

**Published:** 2025-08-18

**Authors:** Nicolás Escrivá, Laura Moreno‐Galarraga, Elena Barado, María Gabriela Torres, Alejandro Fernandez‐Montero

**Affiliations:** ^1^ Department of Occupational Medicine Complejo Hospitalario de Navarra Navarra Spain; ^2^ Instituto de Salud Pública y Laboral de Navarra Navarra Spain; ^3^ Department of Pediatrics, Complejo Hospitalario de Navarra Universidad Pública de Navarra Pamplona Spain; ^4^ Navarra Institute for Health Research (IdiSNA) Pamplona Spain; ^5^ Department of Occupational Medicine University of Navarra Navarra Spain

**Keywords:** acute COVID‐19, long covid, post‐COVID functional status scale, SARS‐CoV‐2 Sequelae

## Abstract

**Background and Aims:**

Long COVID (LC) is a condition characterized by the persistence of physical or psychological symptoms after acute SARS‐CoV‐2 infection. While its pathophysiology remains unclear, it is essential to identify acute‐phase risk factors associated with its development. This study aimed to investigate the association between symptoms during acute COVID‐19 and the risk of developing LC, and to evaluate the impact of LC on functional status in a nonhospitalized population.

**Methods:**

A retrospective observational case‐control study was conducted between May 2022 and March 2024 including 434 participants with confirmed SARS‐CoV‐2 infection. Participants were classified as cases (those with LC; *n* = 226) or controls (those without LC; *n* = 208). Data were collected using a structured electronic form, including self‐reported sociodemographic, clinical, and lifestyle information. Severity and number of acute symptoms were recorded. Functional status was assessed using the Post‐COVID functional status (PCFS) Scale. Logistic and linear regression analyses were performed to explore associations, adjusted for potential confounders.

**Results:**

Severe acute COVID‐19 (defined as pneumonia or hospitalization) was associated with a significantly increased risk of LC (adjusted OR = 7.22; 95% CI: 2.79–18.70). Additionally, each additional symptom during the acute phase increased the odds of LC by 52% (adjusted OR = 1.52; 95% CI: 1.35–1.77). Dyspnea and chest pain were the symptoms most strongly associated with LC.

**Conclusion:**

The severity and symptom burden of acute COVID‐19 are strongly associated with the development of LC and with long‐term functional impairment. These findings highlight the importance of early identification and follow‐up in patients with severe initial COVID‐19 symptoms.

## 1 Introduction

The global understanding of COVID‐19, the disease caused by SARS‐CoV‐2 continues expanding. Beyond the initial impact of this illness, which has resulted in approximately 7 million deaths [1], new public health challenges are emerging. These challenges arise from the persistence of symptoms in a significant percentage of survivors, estimated to impact 6.2% (2.4%–13.3%) [2].

The Centers for Disease Control and Prevention (CDC) and the World Health Organization (WHO) define Long COVID (LC) as: “a broad spectrum of symptoms (physical or mental) that start during or after the acute illness and persist for more than 2 months after the acute process (3 months after the onset of the illness), impact on the patient's life, and are not explained by alternative diagnoses” [3, 4].

Despite the heterogeneity in the definition of LC [5], “there seems to be a consensus on the main symptoms of the acute infection” [4, 6, 7] as well as “those that most frequently persist over time, such as dyspnea, fatigue, headache, and cardiologic or neurologic symptoms, among others” [8].

Due to the increasing number of COVID‐19 survivors requiring follow‐up and aimed to quantify its impact on patients' functional status, the “Post‐COVID‐19 Functional Status Scale (PCFS)” was developed. Klok et al. [9] proposed a tool to follow symptoms and functional status. Similarly, Sepulveda et al. [10] concluded that individuals with LC reporting high PCFS scores reported lower quality of life in functional capacity, pain, and social domains, compared to those with fewer limitations. The scale has been widely used, mostly in hospital‐based cohorts [11, 12, 13, 14].

The first systematic review and meta‐analysis [15] found heterogeneity among the studies. Among the 13,340 patients included, two factors were identified: being female and having a severe clinical condition during hospitalization, which increased the risk of LC. However, the authors emphasize that these results should be interpreted with caution, as they were obtained by combining crude estimates. They also report that one of the most common limitations was the absence of a control group. Subsequent studies confirmed these findings and linked the risk of LC to conditions like hypertension, chronic lung disease, obesity, diabetes, and depression [16, 17, 18, 19].

In this observational case‐control study, we aimed to determine the likelihood of developing LC and its influence on the “PCFS”. In addition, we aimed to investigate its correlation with symptoms experienced during the acute phase of the infection.

Based on current evidence, we hypothesize that the presence of certain symptoms during the acute phase of COVID‐19 is associated with a higher probability of developing LC. Furthermore, we hypothesize that individuals who develop LC will present significantly higher levels of functional limitation, as measured by the PCFS Scale, compared to those who do not develop LC. The primary outcomes of the study are: (1) the presence of LC, and (2) the functional status of patients as measured by the PCFS scale. Secondary outcomes include the identification of acute‐phase symptoms most strongly associated with the development of LC.

## 2 Methodology

### 2.1 Study Definition

The present study is a retrospective, observational, case‐control study that began on May 17, 2022, and remains ongoing. Cases were participants who reported symptoms consistent with a diagnosis of LC. Controls were participants who had experienced acute SARS‐CoV‐2 infection without presenting LC symptoms. Data collection was conducted via an electronic form with self‐reported values, including sociodemographic, clinical, and lifestyle variables. The study design and reporting followed the STROBE (Strengthening the Reporting of Observational Studies in Epidemiology) guidelines to ensure transparency and scientific rigor. Confidentiality and data protection were managed by RedCap® database.

### 2.2 Population Definition

Participants were recruited from the general outpatient population over the age of 5, as well as from members of Long COVID associations. The only requirement for participation was clinical documentation of a prior acute SARS‐CoV‐2 infection. All participants were required to provide laboratory‐confirmed evidence of SARS‐CoV‐2 infection, through either a PCR or antigen test. Cases based solely on clinical diagnosis without a positive test were not included in the study.

**Figure 1:**
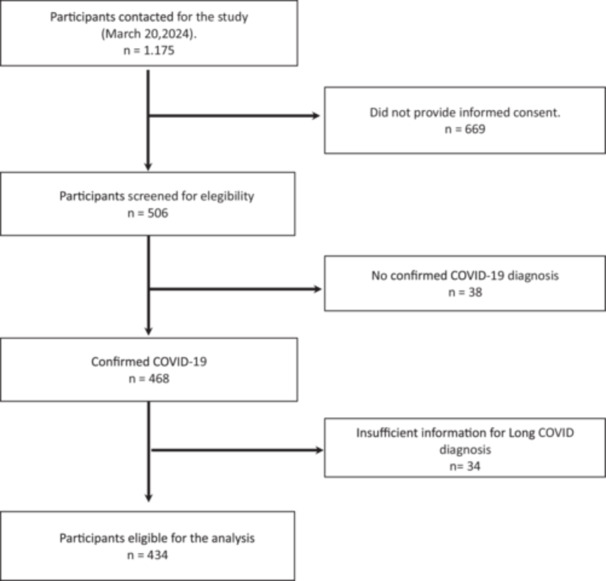
Flow‐chart. Flow diagram showing the selection process of participants included in the retrospective case‐control study. From 1,175 initial participants, exclusions were applied for lack of diagnostic confirmation or missing data, resulting in a final sample.

A total of 1,175 participants who had requested information about the study were collected on March 20, 2024. Figure 1 shows the flow chart. Participants who had not given informed consent, those who had completed the survey but did not have at least one confirmed diagnosis of COVID‐19, or those who did not provide sufficient information to confirm or rule out the presence of LC were excluded. Through this process, a total of 434 participants were finally included in this study.

### 2.3 Main Independent Variables

The independent variables of the study were the symptoms presented during acute SARS‐CoV‐2 infection, as well as the duration, number of symptoms and severity of infection. This information was collected through a multiple‐choice table, in which participants could select symptoms from a list including cough, sore throat, runny nose, dyspnea, pneumonia, fatigue, myalgia, loss of smell, loss of taste, fever, digestive symptoms (vomiting and diarrhea) and dizziness. If any symptoms were selected, participants were asked to specify their duration. Additionally, participants had the option to report symptoms not included in the list.

As for the severity of the infection determined by the presence of pneumonia (diagnosed by imaging tests) or hospital admission to the ward or ICU, the same process was followed as with the symptoms; once the option was selected, participants were required to indicate the duration of hospital stay or ICU admission.

### 2.4 Outcome Variables

The definition of the outcome variable is based on the WHO criteria for Long COVID, defined as a condition occurring in individuals with a history of SARS‐CoV‐2 infection, 3 months after diagnosis of COVID‐19, persisting for at least 2 months and not explainable by any alternative diagnosis [3]. The survey collects the symptoms most frequently associated with LC in published studies on the subject (asthenia, respiratory distress, pneumonia, dizziness, anxiety, depression, sleep disorders, muscle aches, fever, cardiac symptoms, dysgeusia, vomit, headache, chest pain) [20]. However, a free text field was added so that participants could include other symptoms not listed. This is based on the limitations suffered by the patient in daily life in base to “PCFS” [12] and is divided into five categories, ranging from grade 0 (no limitations) to grade 4 (severe limitations requiring assistance), based on how symptoms interfere with daily activities.

### 2.5 Others

Variables related to the status before acute SARS‐CoV‐2 infection were also collected. Sociodemographic variables included age, sex, country of origin, ethnicity, educational level, and employment as a healthcare worker. Regarding personal medical history before the acute infection, participants were asked about pre‐existing diseases and treatments. About lifestyle habits before acute infection, participants were asked about tobacco (smoker, former smoker or Nonsmoker) and alcohol consumption (grams/day), exercise/sedentary habits, diet quality measured with Medas Score [21], sleep hours (MET/h/week), and BMI. In addition, vaccination status at the time of acute illness was recorded (dose, type of vaccine and date), as well as the date of diagnosis and of reinfection if any.

### 2.6 Statistical Analysis

Data were collected using REDCap (Research Electronic Data Capture) hosted at Universidad DE Navarra, a secure, web‐based application designed for research data collection and management, compliant with HIPAA regulations for human subject research data protection.

Categorical variables were expressed as percentages and continuous variables as means and their standard deviation (SD) and associations with continuous outcomes were described using beta (β) coefficients, representing the average change in the dependent variable per unit increase in the predictor.

To assess the association between the severity of COVID‐19 infection and the risk of developing LC, a crude, age‐ and sex‐adjusted and multivariable‐adjusted regression was performed. The multivariable model adjusted for race, flu vaccination, university studies, marital status, employment as a health worker, tobacco use, illness before diagnosis, and prior COVID‐19 vaccination. Results were expressed as odds ratios (OR) with corresponding 95% confidence intervals (CI) to indicate the precision of the estimates.

For missing values of the adjustment variables, a simple imputation was performed using all the adjustment variables, the main independent variable and the outcome variable. The following variables were imputed: flu vaccine (15% of values), university studies (21.4%), illness before COVID‐19 diagnosis (21.9%), pre‐infection vaccine, COVID‐19 vaccination status before infection (4.6%), BMI (27%), alcohol consumption (21.9%), hours of sleep (25.6%), hours of sedentary lifestyle (23%), exercise (23.5%) and smoking status (21.7%).

To assess the association between COVID‐19 symptoms during acute infection and PCFS scale (in participants who reported having LC) a logistic regression and a linear regression adjusted for the same multiple variables were performed, expressing the result in beta coefficient and 95% CI. To assess the relationship between each of the symptoms of COVID‐19 infection and the risk of presenting LC (in participants who reported having LC), a multi‐adjusted logistic regression was also performed with the same variables.

All p‐values are two‐tailed. Statistical significance was considered for a *p*‐value < 0.05. Data processing and analysis were performed using the statistical software Stata® 17.0 version.

During the preparation of this study, the authors used ChatGPT, OpenAI to improve the clarity of the manuscript's language. After using this tool, the authors reviewed and edited the content as needed and takes full responsibility for the content of the publication.

The study was approved by the Ethics Committee of the University of Navarra (Ref. 2023.042), complies with the 2023 Medical Data Protection Law, and follows the Helsinki Declaration.

## 3 Results

The study included 434 participants, all of whom had COVID‐19. Of these, 208 (47.93%, 208/434) did not develop LC (controls), while 226 (52.07%, 226/434) did (cases). The mean age was 43.08 (6.42) years for controls and 44.69 (12.20) years for cases. The percentage of women was higher in both groups: 82.2% (171/208) in controls and 89.3% (186/208) in cases. Table 1 presents the baseline characteristics before acute infection, according to the acute COVID‐19 severity, (severe vs. mild). It can be observed that 17.51% (76/434) of participants had severe COVID‐19 while 82.49% (358/434) had mild COVID‐19. The percentage of women was higher in both groups, although the differences were not significant. However, significant differences were observed in the percentage of participants with university studies, previous influenza vaccination, number of infections and hospital admissions. It is worth noting that the group with severe COVID‐19 presented a lower percentage of participants vaccinated against SARS‐CoV‐2 before infection. This could represent a significant source of bias given the known protective effect of vaccination against severe forms of the disease and potential sequelae. However, this variable was included in the multivariable logistic regression models to control for its confounding effect. Although the severe COVID‐19 group reported fewer daily sedentary hours, they exhibited a significantly higher BMI. This may reflect the presence of underlying metabolic comorbidities or reduced baseline functional capacity, which are not always captured by self‐reported sedentary time alone.

**Table 1:** Basal characteristics of study participants (*n*: 434) before acute infection, presented according to acute COVID‐19 severity.

Variable	Mild COVID‐19	Severe COVID‐19^a^	*p*
Number, *n* (%)	358 (82.49)	76 (17.51)	
Age, (years), mean (SD)	42.96 (49.74)	48.43 (10.09)	*p* = 0.34
Sex, woman, %	82.96% (297/358)	78.95% (60/76)	*p* = 0.41
Marital status, married or in a domestic partnership, %	55.31% (198/358)	55.26% (42/76)	*p* = 0.19
Race, %			*p* = 0.23
Caucasic	90.22% (323/358)	86.84% (66/76)	
Latin‐American	7.82	10.53	
Other	1.96	2.63	
University studies, %	82.80% (296/358)	67.74% (52/76)	*p* = 0.007
Employment status, %			*p* = 0.39
Worker	65.92	73.68	
Unemployed	12.01	7.89	
Health care worker, %	10.89% (39/358)	10.53% (8/76)	*p* = 0.19
Previous diseases	36.46	51.61	*p* = 0.03
Tobacco use, %			*p* = 0.03
Smoker	14.03% (50/358)	8.06% (6/76)	
Former smoker	5.76% (21/358)	14.52% (11/76)	
Nonsmoker	80.22% (287/358)	77.42% (59/76)	
Previous influenza vaccination, %	48.36% (173/358)	43.08% (33/76)	*p* = 0.44
Previous COVID‐19 vaccination, %	41.06% (147/358)	12.33% (10/76)	*p* < 0.001
BMI^b^, mean (SD)	23.24 (3.90)	27.47 (5.19)	*p* < 0.001
Alcohol intake^c^, mean (SD)	2.12 (2.89)	2.37 (3.79)	*p* = 0.57
Sleep hours per day, mean (SD)	7.63 (1.07)	7.54 (1.01)	*p* = 0.58
Sitting hours per day, mean (SD)	6.87 (3.05)	5.99 (2.98)	*p* = 0.04
Physical activity^d^	26.33	32.26	*p* = 0.06
Adherence to Mediterranean diet^e^	6.82	6.58	*p* = 0.44

*Note:* Values have been calculated based on the number of valid responses.

Abbreviations: BMI, body mass index; ICU, intensive care unit; N, number; SD, standard deviation.

^a^ Severe COVID‐19: pneumonia, UCI or hospitalization.

^b^ Expressed in kg/m^2^.

^c^ Expressed in grams/day.

^d^ Expressed in METs/week.

^e^ Medas Score: mediterranean diet adherence screener.

Table 2 shows the association between having severe COVID‐19 infection or not and the risk of LC. Participants who had severe COVID‐19 infection had an OR = 7.22 CI 95% (2.79–18.7) compared to participants who had mild symptoms. When we analyzed the risk for each symptom of having LC for each symptom that the patient added, it was seen that the risk increased 1.52 times CI 95% (1.35–1.77) of having severe COVID‐19.

**Table 2:** Logistic regression. Risk of developing Long COVID based on vaccination status before COVID‐19 infection.

Variable	Mild symptoms or asymptomatic	Severe symptoms (pneumonia, hospitalization, or ICU)	CI 95%	Per each symptom	CI 95%
*N*	358	76			
Case	158	68			
Crude OR	1 (ref.)	10.76	(5.02–23.05)	1.63	(1.48–1.80)
Sex, age‐adjusted OR	1 (ref.)	10.80	(5.04–23.15)	1.64	(1.48–1.81)
Multivariable‐adjusted OR^a^	1 (ref.)	7.22	(2.79–18.7)	1.52	(1.35–1.77)

Abbreviations: CI, confidence interval; OR, odds ratio.

^a^ Adjusted for sex, age, university studies, body mass index, physical activity, race, tobacco use, alcohol intake, adherence to Mediterranean diet, previous illness, flu vaccination, health care worker, high risk of COVID worker, sleep hours, sedentarism and vaccination status.

**Figure 2:**
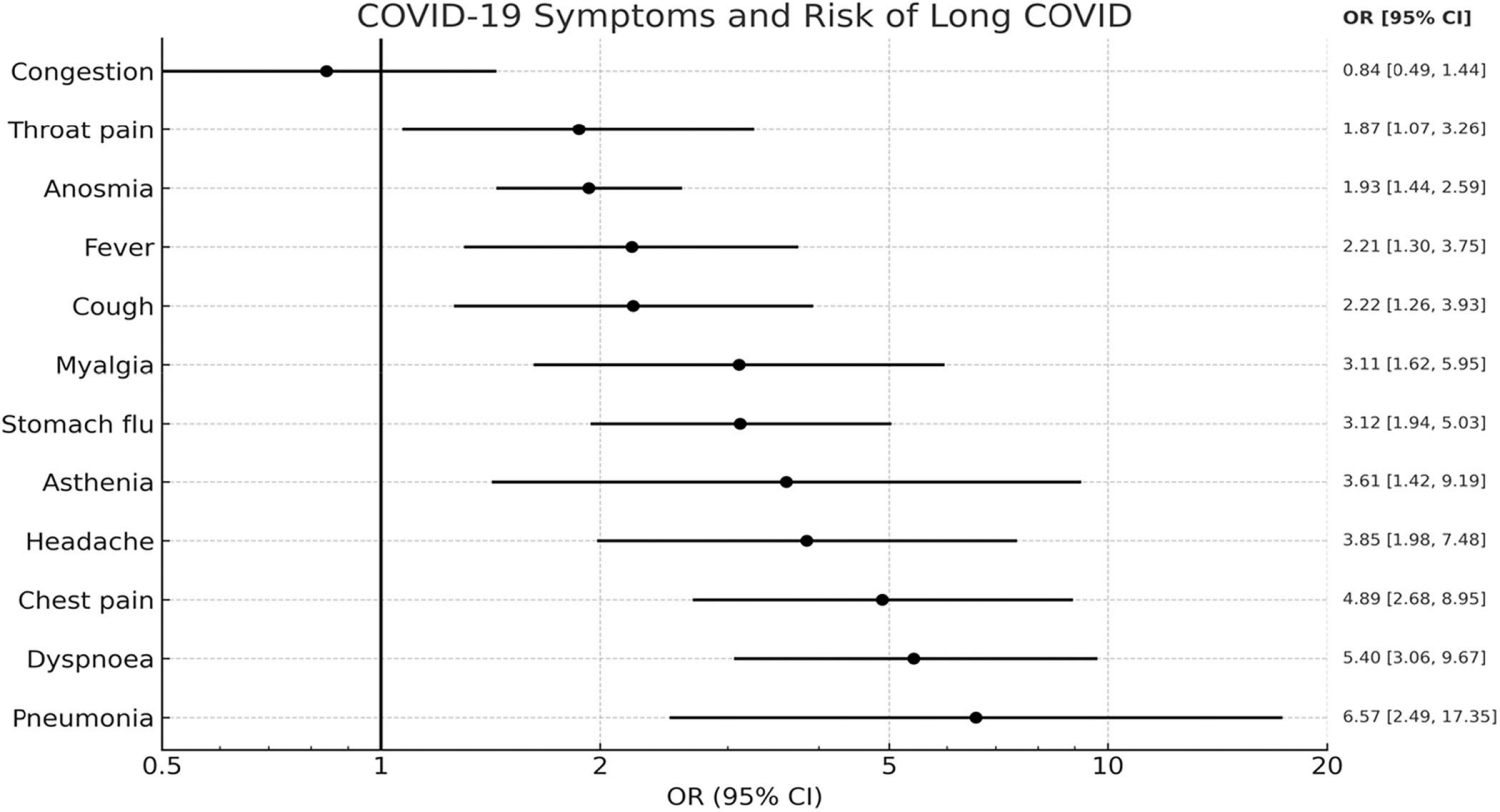
COVID‐19 symptoms and Risk of LC. Forest plot showing the association between individual symptoms during acute SARS‐CoV‐2 infection and the odds of developing Long COVID (LC) in the study population (*n* = 434). Odds ratios (OR) and 95% confidence intervals (CI) were derived from multivariable logistic regression adjusted for age, sex, BMI, comorbidities, and vaccination status. Symptoms significantly associated with LC include dyspnea, chest pain, and fatigue.

In Figure 2, each of the symptoms of COVID‐19 infection is analyzed with respect to the risk of suffering from LC we see that flu, dyspnea, chest pain, dyspnea, are the symptoms with the highest risk of presenting LC.

**Figure 3:**
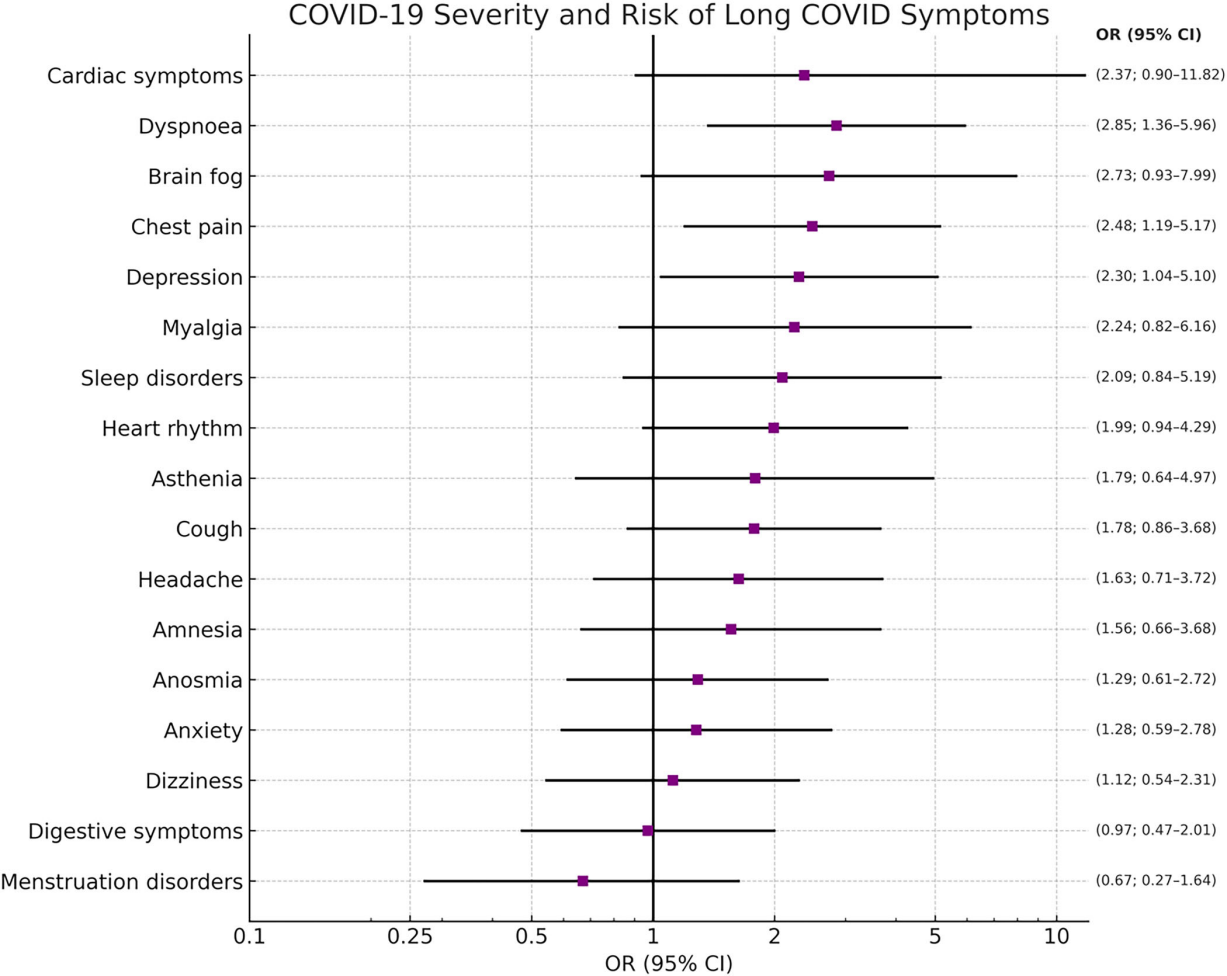
COVID‐19 severity and risk of LC. Forest plot showing the odds ratios (OR) and 95% confidence intervals (CI) for the association between acute COVID‐19 severity and the presence of persistent symptoms.

Figure 3 shows the relationship between the severity of acute COVID‐19 infection and each of the symptoms of LC. It can be observed that depression, chest tightness and dyspnea are associated in a statistically significant way.

**Figure 4:**
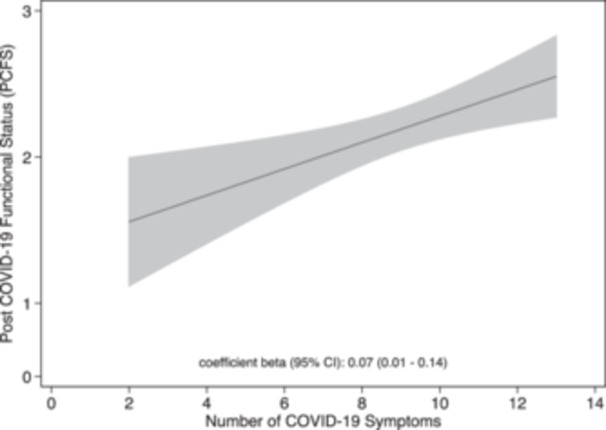
Relationship between Post‐COVID‐19 Functional Status (PCFS) scale and the number of COVID‐19 symptoms during acute infection. (*) PCFS: 0: no functional limitation; 1: nonsignificant functional limitation; 2: mild functional limitation; 3–4: moderate or severe functional limitation.

Figure 4 illustrates the relationship between the Post COVID‐19 Functional Status (PCFS) scale and the number of symptoms experienced during acute COVID‐19 infection, with a Beta coefficient (95% CI) of 0.07 (0.01–0.14). The figure shows that as the number of symptoms increases, functional status worsens, indicating greater functional limitations with a higher symptom burden.

## 4 Discussion

Our study provides key insights into the factors influencing the development of LC and its impact on patients' functional status. We found that the severity of the acute infection, specific symptoms, and the total number of symptoms during the acute infection are significant predictors of LC.

We analyzed the relationship between acute infection symptoms and the risk of developing LC and the relationship between the severity of acute infection and each LC symptoms. In agreement with previous studies, such as the 2021 meta‐analysis [15] this study finds a statistically significant association between the severity of acute COVID‐19 and the risk of developing LC, with a higher prevalence of LC in patients who presented severe forms of the disease. As seen in other studies [7, 8], acute symptoms such as dyspnea and chest pain are associated with the development of LC. This supports the idea that certain symptoms may indicate a more severe inflammatory response or a higher viral load, which could predispose patients to long‐term complications. This inflammatory response may persist after acute infection and could result in immune system dysregulation, leading to prolonged persistent symptoms and complications of COVID‐19 infection [22, 23]. While p‐values indicated statistical significance, we focused on reporting odds ratios with 95% confidence intervals to reflect the magnitude and precision of the associations. This approach aligns with current recommendations for clinical data reporting.

The PCFS showed strong associations with health‐related quality of life (HRQoL) and functional limitations, confirming its construct validity among symptomatic COVID‐19 patients [18]. Our study demonstrates a correlation between the increase in the PCFS and the number of symptoms experienced during acute infection, showing that individuals with more COVID‐19 symptoms tend to have greater functional limitations as measured by the PCFS Scale. In our study, individuals with more severe acute COVID‐19 presented a higher BMI and reported lower levels of sedentary behavior—an apparently contradictory finding. This may reflect the complexity of self‐reported lifestyle data and suggests that metabolic factors, rather than physical inactivity alone, could play a more relevant role in disease severity. Given that greater severity during the acute phase is strongly associated with long COVID, these results underline the importance of considering underlying metabolic health when assessing LC risk. Our findings align with previous reports, such as those by Xie et al. [7] and Natarajan et al. [8], which showed similar associations between acute dyspnea and LC development. However, those previous studies primarily included patients admitted to hospitals. It is well known that hospital stays can potentially affect the immune response and the body's ability to recover from infection, potentially increasing the likelihood of developing LC symptoms in these patients [24]. However, our study is distinguished by focusing on a nonhospital population, allowing for a more representative perspective of the general population.

One difference observed compared to some studies is that while fatigue is often reported as the most frequent symptom of LC, it did not appear as the most prevalent symptom in this study. This discrepancy could be attributed to differences in data collection methods (self‐reported vs. clinical assessment) or variations in how participants defined their symptoms.

Another key difference between this study and many previous ones is that earlier research has focused on hospitalized populations, which could have introduced bias due to the inherently more severe nature of those cases. Patients requiring hospitalization often present with more acute and life‐threatening symptoms, leading to a higher likelihood of long‐term complications such as LC. As a result, findings from these studies may overestimate the prevalence and severity of LC by focusing on a subset of the population that experienced more severe infections.

In contrast, our study focuses on a general nonhospitalized population, which provides a broader and potentially more representative view of LC's impact in the general population. By including individuals with milder or moderate infections who were not hospitalized, our findings may better reflect the true spectrum of LC symptoms and their prevalence in the wider community. This approach allows us to capture cases that might have been underrepresented in studies restricted to hospitalized patients, offering new insights into how LC affects individuals with less severe acute COVID‐19 infections and highlighting the widespread nature of long‐term complications across different levels of disease severity.

Our study has several strengths and limitations that should be acknowledged. These factors are important to consider when interpreting the findings and their implication. Regarding its strengths, this study stands out for analyzing in detail the relationship between the severity of acute COVID‐19 infection and the subsequent development of LC with a comprehensive approach. Multiple predictor variables are included, such as the number of acute symptoms, disease severity and specific symptoms. We consider this study to make a key contribution, as much of the previous research has focused on hospitalized populations, whereas our study includes a broader and more representative sample. In terms of limitations, despite the robustness of the analysis, the sample size can be considered limited compared to larger studies or previous meta‐analyses. This may affect the ability to generalize the findings to a broader population. A larger sample size could have allowed for greater precision. Regarding the participants, a possible selection bias, since the study focuses on patients from LC associations. In a retrospective case‐control study, sometimes it's difficult to establish a causal relationship because both the exposure and the outcome have already occurred at the time of data collection. Another possible limitation is recall bias: patients who develop LC may be more likely to remember their acute symptoms in a more severe manner, which could influence the reported relationship between initial severity and the presence of LC. On the other hand, although several predictors of LC were analyzed, it is possible that some confounding factors, such as unmeasured comorbidities, were not fully controlled. It is also important to consider that a considerable portion of participants who experienced severe acute COVID‐19 were infected during the early waves of the pandemic, before vaccines became available. This temporal aspect may explain the lower vaccination rates observed in the severe group and helps contextualize this finding. While this could introduce a potential bias, vaccination status was included as an adjustment variable in the multivariable analysis to minimize confounding.

Given the findings of this study, it would be advisable for healthcare professionals to consider a more proactive approach in monitoring patients who experienced severe symptoms during the acute phase of COVID‐19. The severity of the infection and the number of symptoms appear to be related to a higher risk of developing Long COVID. Careful monitoring after the initial recovery could facilitate the early detection of persistent symptoms, which could potentially help mitigate their long‐term impact. Additionally, in postinfection consultations, it would be prudent for physicians to comprehensively assess the symptom burden, particularly in cases of dyspnea and chest pain, which might be associated with prolonged complications. The use of tools such as the Post‐COVID Functional Status Scale could be helpful in identifying potential functional limitations and guiding decision‐making regarding interventions that may improve patients' quality of life.

## 5 Conclusions

In conclusion, this study identifies severity of acute illness and symptom burden as key predictors of Long COVID in nonhospitalized individuals, highlighting the need for early follow‐up strategies and the use of tools such as PCFS in primary care. It shows that severe acute COVID‐19 increases the risk of LC, consistent with previous findings, and suggests that specific symptoms like dyspnea and chest pain may indicate a stronger inflammatory response.

The study also highlights the link between acute symptoms and functional limitations, measured by the PCFS. However, limitations such as the small sample size, potential selection bias, and recall bias should be considered. Further research with larger and more diverse populations is needed to strengthen these findings.

## Author Contributions

**Nicolás Escrivá:** writing – review and editing, writing – original draft, investigation, conceptualization, methodology, software. **Laura Moreno‐Galarraga:** conceptualization, investigation, writing – original draft, writing – review and editing, methodology, validation, visualization, supervision, data curation, project administration, software. **Elena Barado:** investigation, methodology. **María Gabriela Torres:** investigation, conceptualization, methodology, validation. **Alejandro Fernandez‐Montero:** conceptualization, investigation, writing – original draft, methodology, validation, visualization, writing – review and editing, project administration, supervision, data curation, software.

## Ethics Statement

the study was approved by the Ethics Committee of the University of Navarra (Ref. 2023.042), complies with the 2023 Medical Data Protection Law, and follows the Helsinki Declaration.

## Conflicts of Interest

The authors declare no conflicts of interest.

## Transparency Statement

The corresponding author Dr. Laura Moreno‐Galarraga affirms that this manuscript is an honest, accurate, and transparent account of the study being reported; that no important aspects of the study have been omitted; and that any discrepancies from the study as planned (and, if relevant, registered) have been explained.

## Supporting information


**Supplementary table 1** represents the data related to the baseline characteristics based on Cases and controls.

## Data Availability

The data that support the findings of this study are available from the corresponding author upon reasonable request. The data and materials supporting the findings of this study are available upon request from the corresponding author (laura.moreno.galarraga@navarra.es). The data will be shared in accordance with applicable regulations, privacy concerns and ethical guidelines.
