# Supplementary material for: Association Between Acute COVID‐19 Infection and Long COVID in a Non‐Hospitalized Population: A Retrospective Case‐Control Study
Source: Health Sci Rep. 2025 Aug 18;8(8):e71043. doi: 10.1002/hsr2.71043 (PMC12361630; doi:10.1002/hsr2.71043)
Supplement: Supplementary file 1 — Supplementary table 1 represents the data related to the baseline characteristics based on Cases and controls. [file HSR2-8-e71043-s001.docx]

|  | | | |
| --- | --- | --- | --- |
| **Supplementary Table 1: baseline characteristics of study participants (n: 434) based on Cases and controls** | | | |
|  |  |  |  |
| **Variable** | **Controls** | **Cases** | **P** |
|  |  |  |  |
| Number, n (%) | 208 (47.93%, 208/434) | 226 (52.07%, 226/434) |  |
| Age, (years), mean (SD) | 43.08 (6.42) | 44.69 (12.20) | P=.71 |
| Sex, woman, % | 82.2% (171/208) | 89.3% (186/208) | P=.98 |
| Marital status, married or in a domestic partnership, % | 55.77 | 54.87 | P=.85 |
| Race, % |  |  | P=.30 |
| - Caucasic | 88.46 | 90.71 |  |
| - Latin-American | 10.10 | 6.64 |  |
| - Other | 1.44 | 2.65 |  |
|  |  |  |  |
| University studies, % | 85.03% (177/208) | 76.29% (172/226) | P=.05 |
| Employment status, % |  |  | P=.13 |
| - Worker | 82.31 | 88.14 |  |
| - Unemployed | 17.69 | 11.86 |  |
|  |  |  |  |
| Health care worker, % | 48.30% (100/208) | 24.74% (56/226) | P=<.001 |
| Previous diseases | 28.97 | 46.91 | P<.001 |
| Tobacco use, % |  |  | P=.64 |
| - Smoker | 14.38% (30/208) | 11.86% (27/226) |  |
| - Former smoker | 6.16% (13/208) | 8.25% (18/226) |  |
| - Non-smoker | 79.45% (165/208) | 79.90% (181/226) |  |
|  |  |  |  |
| Previous influenza vaccination, % | 52.76% (110/208) | 43.20% (97/226) | P=.07 |
| Previous COVID-19 vaccination, % | 45.92% (95/208) | 27.06% (61/226) | P=<.001 |
| BMI ^(*)^, mean (SD) | 22.70 (3.50) | 24.86 (4.80) | P<.001 |
| Alcohol intake ^(ϑ)^, mean (SD) | 2.30 (2.53) | 2.07 (3.42) | P=.49 |
| Sleep hours per day, mean (SD) | 7.62 (1.00) | 7.60 (1.11) | P=.84 |
| Sitting hours per day, mean (SD) | 7.07 (2.84) | 6.44 (3.18) | P=.059 |
| Physical activity ^(§)^ | 21.42 (14.75) | 31.87 (25.28) | P=<.001 |
| Adherence to Mediterranean diet ^(ε)^ | 6.26 (2.08) | 7.15 (2.20) | P=<.001 |
|  | | | |
|  | | | |
| Note. Values have been calculated based on the number of valid responses. N: number. BMI: body mass index. ICU: intensive care unit. SD: standard deviation.  (*) Severe COVID-19: ICU or pneumonia; (ϕ) Expressed in kg/m2; (§) Expressed in METs/week; (ϑ); Expressed in grams/day; (ε) Predimed Score (< 9 low adherence to the Mediterranean diet, >= 9 good adherence to the Mediterranean diet). | | | |
